# Simultaneous Quantification of Protein Binding Kinetics in Whole Cells with Surface Plasmon Resonance Imaging and Edge Deformation Tracking

**DOI:** 10.3390/membranes10090247

**Published:** 2020-09-22

**Authors:** Wenwen Jing, Ashley Hunt, Nongjian Tao, Fenni Zhang, Shaopeng Wang

**Affiliations:** 1 Biodesign Center for Bioelectronics and Biosensors, Arizona State University, Tempe, AZ 85287, USA; wjing5@asu.edu (W.J.); ashley@biosensinginstrument.com (A.H.); Nongjian.Tao@asu.edu (N.T.); 2 School of Molecular Sciences, Arizona State University, Tempe, AZ 85287, USA; 3 School of Electrical, Energy and Computer Engineering, Arizona State University, Tempe, AZ 85287, USA

**Keywords:** binding kinetics, surface plasmonic resonance, cell edge tracking, whole cell, wheat germ agglutinin

## Abstract

Most drugs work by binding to receptors on the cell surface. Quantification of binding kinetics between drug and membrane protein is an essential step in drug discovery. Current methods for measuring binding kinetics involve extracting the membrane protein and labeling, and both have issues. Surface plasmon resonance (SPR) imaging has been demonstrated for quantification of protein binding to cells with single-cell resolution, but it only senses the bottom of the cell and the signal diminishes with the molecule size. We have discovered that ligand binding to the cell surface is accompanied by a small cell membrane deformation, which can be used to measure the binding kinetics by tracking the cell edge deformation. Here, we report the first integration of SPR imaging and cell edge tracking methods in a single device, and we use lectin interaction as a model system to demonstrate the capability of the device. The integration enables the simultaneous collection of complementary information provided by both methods. Edge tracking provides the advantage of small molecule binding detection capability, while the SPR signal scales with the ligand mass and can quantify membrane protein density. The kinetic constants from the two methods were cross-validated and found to be in agreement at the single-cell level. The variation of observed rate constant between the two methods is about 0.009 s^−1^, which is about the same level as the cell-to-cell variations. This result confirms that both methods can be used to measure whole-cell binding kinetics, and the integration improves the reliability and capability of the measurement.

## 1. Introduction

Receptor–ligand reactions are central to much of the field of pharmacology, and more than 60% of drugs on the market target at cell membrane proteins [1]. However, there are several major challenges when studying membrane protein binding kinetics. Extraction is a tedious, laborious task of physically separating the membrane protein from its membrane environment [2]. The conformation of the membrane protein may also change due to the detergents [3,4]. These issues plague the study of membrane protein outside the membrane. Fluorescence labeling is commonly used, but it is not feasible for small molecules, which poses a problem, particularly to pharmacology, since nearly 90% of drugs are small molecules [5]. Radiolabeling works without affecting binding kinetics, even with small molecules, but it is extremely costly and involves the hazards of radioactive materials. It also demands custom synthesis of reagents and has insufficient time resolution to measure fast reactions. Förster resonance energy transfer (FRET) and chemiluminescence can achieve continuous monitoring but requires a carefully designed reaction scheme [5]. While surface plasmon resonance (SPR) has been used for measuring binding kinetics on whole cells and quantifying receptor density on cell surface [6], it is incapable of detecting binding events higher than 100 nm above the surface [7]. In addition, the SPR signal is scaled with the size of ligand molecules, and it is challenging to detect small molecules binding to large membrane receptors. We have developed an edge tracking method that detects nanometer-scale changes in cell shape accompanied by the membrane protein binding events for label-free kinetic quantification that is capable of measuring small molecule binding kinetics to membrane receptors on the whole-cell surface [8,9].

Here, we report the integration of the edge tracking method to a dual-mode SPR imaging system for simultaneous measurement of the kinetics of ligand–receptor binding on whole cells with both edge tracking and SPR. This integration not only provides the complementary capabilities of both methods on a single device but also allows a direct comparison between these two methods on binding kinetic measurement at the single-cell level. Wheat germ agglutinin (WGA), which binds primarily to the sugar N-acetyl-D-glucosamine that attaches to glycoproteins on the cell surface, was used as a model system. Since glycoproteins are abundant on all cell membranes, WGA can be used as a universal model ligand for all cell lines. Our results show that the kinetic constants obtained from the two orthogonal methods are consistent for individual cells. This integrated solution combines the strength of the two methods and provides a reliable label-free method to quantify membrane protein binding kinetics on whole cells, with detection range extendable to small-molecule ligands.

## 2. Materials and Methods

### 2.1. Cell Culture

All cell culturing protocols were done inside a biological safety cabinet (BSC). The SH-EP1_α4β2 cell line was engineered from the SH-EP1 cell line (ATCC, Manassas, VA, USA). The cells were grown in DMEM (Lonza, Basel, Switzerland) supplemented with 10% FBS (Thermo Fisher Scientific, Waltham, MA, USA) and 40 units/mL penicillin and 40 µg/mL streptomycin sulfate (Thermo Fisher Scientific, Waltham, MA, USA) in 25 cm^2^ flasks (VWR, Radnor, PA, USA). Cells were passaged every 2 to 3 days, whenever 80% confluency was reached. Cells were briefly washed when passaging with 5 mL Hank’s Buffered Salt Solution (Thermo Fisher Scientific, Waltham, MA, USA). Then, 2 mL of 0.05% trypsin–EDTA (Thermo Fisher Scientific, Waltham, MA, USA) was added, and the flask was incubated at 37 °C for approximately 1 min until the cells started to detach. The flask was then returned to the BSC and was rinsed with 3 mL of medium. The solution was then centrifuged at 900 rpm for 5 min to form a cell pellet. The liquid was removed, and the cells were suspended in 1 mL of medium, giving a concentration of roughly 10^5^ cells/mL. A passage ratio of 1:5 or 1:7 was used to seed a new flask.

### 2.2. Sensor Chip Preparation

Briefly, 22 × 22 mm gold-coated glass coverslips were rinsed with ethanol, followed by water, and blow-dried with nitrogen gas. A FlexiPERM Silicon chamber (ARSTEDT AG & Co., Nümbrecht GERMANY) with a volume of 800 µL was cleaned with the same protocol and pressed onto the gold chips. Chips with wells were sterilized by exposing them to UV light for 5 min in the BSC. Type IV collagen (Sigma Aldrich, St Louis, MO, USA) was diluted to 10 µg/mL in phosphate-buffered saline (PBS, VWR, Radnor, PA, USA) before being added to the wells. The wells with 700 µL of collagen solution were incubated at 37 °C and 100% relative humidity (RH) for 2 h. The collagen solution was then vacuumed out, and the chips were washed with Hank’s Balanced Salt Solution (HBSS) (Thermo Fisher Scientific, Waltham, MA, USA). Cells were passaged, and the suspended cell solution was 100 times diluted with medium. Then, 700 µL of diluted cell suspension was added to the collagen-modified chips and incubated for 1 h, at which point the medium was vacuumed out and the cells were briefly washed with HBSS. Then, 4% formaldehyde (Santa Cruz Biotechnology, Dallas, TX, USA) in PBS was added for 10 min fixation. Then, the formaldehyde was vacuumed out, and the cells were washed three times with PBS. Finally, 700 µL of PBS was added, and the cells were stored at 4 °C before using. All cells were used within one day of being prepared.

### 2.3. Instrumentation

**Figure 1:**
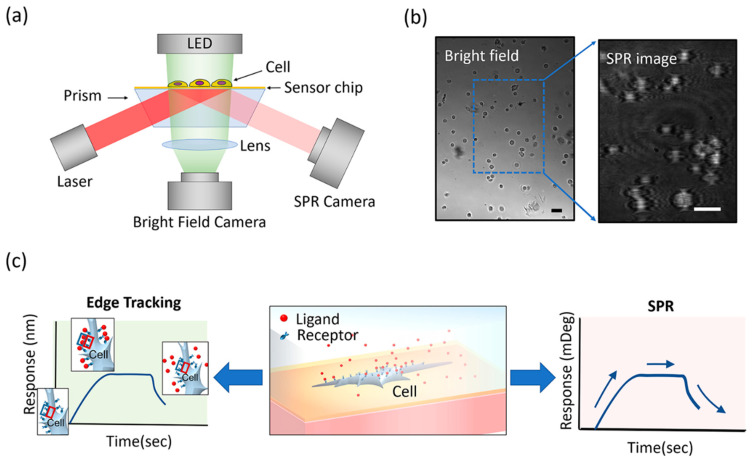
Overview of the experiments. (**a**) Schematic of the setup. Cells are grown and attached to gold chips and placed in a fluidics device where the images are recorded by two cameras. One camera is set up to record surface plasmon resonance (SPR) images, and the other records bright-field images. (**b**) Bright-field and SPR images taken from the instrument. The SPR camera captures only a quarter of the bright field area but provides greater pixel resolution. Scale bar represents 20 µm. (**c**) A zoom-in schematic of one cell to illustrate binding affinity and kinetics analysis simultaneously by the edge tracking method and the SPR method. As the ligand molecules (in this case, lectins) are introduced by the flow system to the cell, binding-induced cell edge deformation occurs. This edge deformation is quantified and compared to simultaneously collected SPR signals.

A recently developed commercial SPR imaging instrument (SPRm 200, Biosensing Instrument Tempe, AZ, USA) was used for all experiments. This is a dual-mode SPR/bright field imaging instrument designed for cell-based binding kinetic measurement. It has 10× optical zoom, a 690 nm 1 mW laser light for SPR imaging and a blue LED top illumination light for bright field imaging, and two CMOS cameras for simultaneous recording of SPR and bright-field images (Figure 1a). The system also has temperature control, microfluidic sample delivery, and an auto-sampler.

### 2.4. Experimental Protocol

The cells prepared, as described above, were measured in the SPRm 200 system. The running buffer was 1× PBS at 250 µL/min. Kinetics experiments involved a short, initial baseline reading, followed by 2 min of association phase when the analyte was introduced to the cells at a given concentration. Then, the solution was switched back to 1× PBS, and the dissociation phase was recorded for another 2 min. All data were recorded at 1 frame per second (fps). Blanks were tested with 1× PBS. SPR sensitivity was calibrated with 0.9× PBS. The ligand, wheat germ agglutinin (36 kDa, Sigma Aldrich, St Louis, MO, USA) with a concentration of 20 and 100 µg/mL, was used for the binding kinetic measurement.

### 2.5. Data Processing

SPR curves were extracted from videos using ImageJ. For calibration, the intensity changes between 1× PBS and 0.9× PBS were set to 23 millidegree (mDeg). A calibration curve was obtained for every sensor chip. Kinetic response curves for WGA binding with glycoproteins were obtained by analyzing the cell edge image intensity in ImageJ. A region of interest (ROI) was selected along the cell edge, and the average intensity change for the region was converted to mDeg and used as a kinetics curve.

The bright-field images were processed with a differential detection algorithm developed inhouse for precise tracking of cell edge deformation; the details of the algorithm were reported in our previous work [8]. Using this method, we can detect the cell edge deformation by calculating the differential image intensity of two rectangular regions of interest (ROIs) at the cell edge, which is defined as (I_1_ − I_2_)/(I_1_ + I_2_), where I_1_ and I_2_ are the intensities of the red and blue ROIs in Figure 1c, respectively. The binding kinetics curve is obtained by plotting the extracted cell edge deformation value over time for the duration of the binding experiment.

By fitting the kinetic curves obtained from edge tracking or SPR with first-order kinetics using Scrubber (BioLogic Software Pty Ltd., Campbell, Australia), the association rate constant ($k_{a}$), dissociation rate constant ($k_{d}$), as well as equilibrium constant ($K_{D}$), are calculated.

## 3. Results and Discussion

### 3.1. Overview of the Integrated Imaging System

The receptor–ligand binding and exocytosis-induced cell deformation have been studied using a high-zoom microscope setup with 40× objectives [8,9,10]. In this study, we test the sensitivity of edge tracking with the 10× optical zoom provided by SPRm 200, which can simultaneously record both SPR images and transmitted images of a dozen cells on one sensor surface (Figure 1a). The system was designed for quantifying binding kinetics on whole cells using SPR imaging, which can also obtain the transmitted images of the cells. The transmitted image allows us to track binding-induced deformation with our edge tracking method and gauge the performance of the edge tracking method with simultaneously recorded SPR data at the single-cell level.

### 3.2. Single Cell Binding Kinetics Measured by Simultaneous Edge Tracking and SPR

A previous edge tracking study reported that the averaged kinetic constants from multiple cells agreed with the literature [9]. However, due to the high cell-to-cell variability, it was hard to gauge how accurate the values were. On top of that, differences in platforms and research groups also introduce discrepancies [11]. Thus, comparing edge tracking measurement at the single-cell level with simultaneously recorded SPR will provide direct and unbiased validation for both methods by removing sources of error mentioned above.

**Figure 2:**
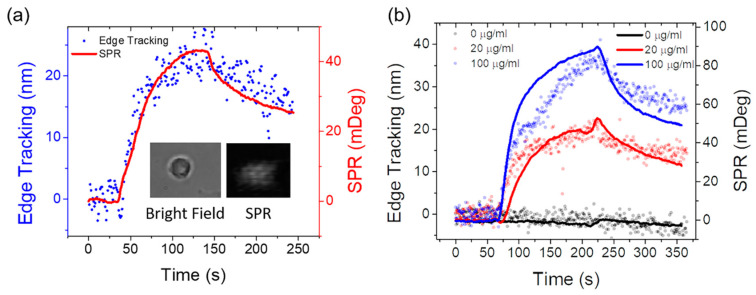
Comparison of both images and kinetic response curves measured by simultaneous edge tracking and SPR imaging on a single cell. (**a**) Simultaneously recorded edge tracking (blue) and SPR (red) response curve of 20 µg/mL wheat germ agglutinin (WGA) binding to an SH-EP α4β2 cell (Inset: bright field (left) and SPR (right) images of the cell). (**b**) Averaged binding response curves of different concentrations of WGA binding to the cells on the chip from the two methods. The solid lines represent SPR results, and the dots represent the edge tracking results.

Figure 2 compares the edge tracking response with the SPR response of WGA binding to the same cell (Figure 2a) and the corresponding dose curve (Figure 2b). The response curves from both methods are well overlaid. The noise level of edge tracking is larger than SPR. This is because the setup is not optimized for edge tracking measurement. Both low optical zoom and slow frame rate contribute to the increased noise level. In optimized setups for edge tracking, the noise is comparable to SPR for this reaction [8,9].

The integration of edge tracking and SPR imaging enables the simultaneous collection of complementary information provided by both methods for molecular interaction detection. SPR signal is known to be scaled with the mass of bound molecules, where it is limited to the detection of small-molecule binding. In contrast, edge deformation is more related to binding-induced surface energy changes [8], which can work with both large- and small-molecule binding [8,9]. However, the edge tracking signal is hard to be interpreted in terms of the bound-molecule quantity, although we do observe that the deformation amplitude is scaled with the number of bound ligands for a given ligand and receptor pair and cell line. It is hard to quantify the receptor density with edge tracking, which can be done with SPR imaging [6]. With the integrated setup, a cross-calibration of edge deformation can be obtained with the simultaneous SPR imaging. In the case of WGA binding, we found the conversion factor between edge tracking and SPR signal to be 3.13 ± 0.77 mDeg/nm.

### 3.3. Variability Validation

**Figure 3:**
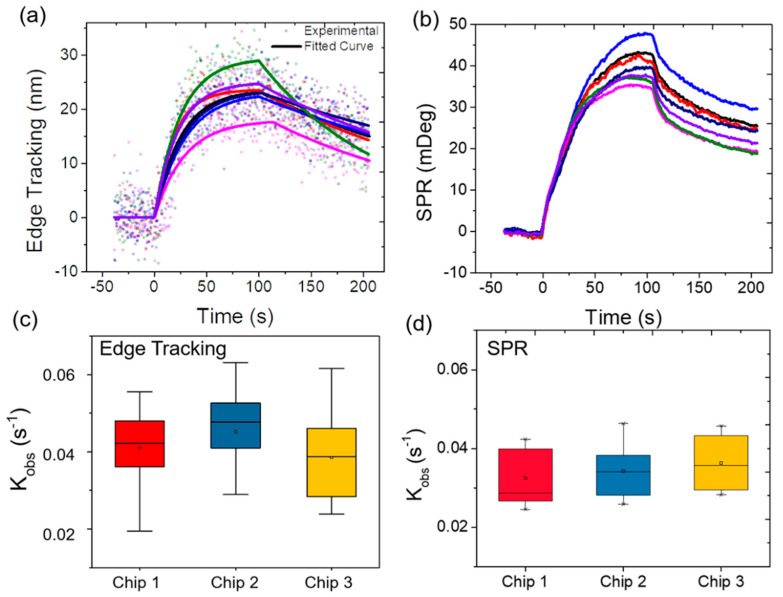
Cell-to-cell and chip-to-chip variability. Binding response curves from different cells on the same chip were recorded simultaneously by (**a**) edge tracking and (**b**) SPR (WGA concentration 20 µg/mL). Dots and solid lines represent experimental data and first-order kinetic fitted curves. Signals from the same cells are shown in the same color. Box and whisker plots of the observed kinetics constant measured by simultaneous (**c**) edge tracking and (**d**) SPR from 3 different chips. Each chip had at least a dozen cells, and all cells were exposed to 100 μg/mL WGA.

WGA binding with glycoproteins is a second-order reaction; however, under continuous flow, it becomes a pseudo first-order reaction. Previous studies have shown that the binding of WGA to cell surfaces is complex [12]. WGA primarily binds to the sugar N-acetyl-D-glucosamine, which is incorporated into many different glycoproteins, each of which can have its own kinetic constant [13,14]. How this sugar is arranged with respect to the rest of the sugars can greatly affect binding [15]. Additionally, WGA is reported to interact with sialic-acid-containing glycoconjugates and oligosaccharides, which may also contribute to the kinetic variations. Considering the complexity of the reaction and the heterogeneity of a cell’s glycocalyx, it is easy to understand why there are obvious cell-to-cell variations (Figure 3a). As shown in Figure 3, cell-to-cell variation was observed for both SPR imaging and edge tracking in both the magnitude and rate of the binding. The binding kinetics from edge tracking and SPR imaging of individual cells were fitted and are shown in Table 1. The kinetic constants obtained from these two methods are consistent for each cell. With the integrated system, the heterogeneity response from cells can be cross-validated among the methods for individual cells, which is more accurate than the previous averaged kinetics comparison.

The box and whisker plots in Figure 3c,d show a comprehensive view of the variation within a chip and between chips for edge tracking and SPR imaging results. Each chip has at least a dozen cells, and different chips were tested on different days. The standard deviations of each chip represent cell-to-cell variation within the chip, and the differences in the mean among the chips represent chip-to-chip variation. The chip-to-chip variation is smaller than the cell-to-cell variation as the difference in mean values is much smaller than the standard deviations of individual chips. This result indicates that while there is high cell-to-cell variation in lectin binding kinetics due to cell surface structure diversity, the integrated system itself is consistent from chip to chip. In addition, regardless of the higher noise level for edge tracking results, neither the mean nor the spread of fitting results differs greatly from the previous edge tracking measurements carried out on high zoom microscopes. Therefore, with some improvement in the bright field image quality, such as increasing the recording frame rate to reduce shot noise and/or increasing optical numerical aperture to improve spatial resolution, the SPRm 200 can be a trusted system to carry out edge tracking detection.

### 3.4. Correlation Analysis of the Observed Kinetics Constant

To further compare the kinetic constants obtained by the two methods, the observed rate constants for different cells were calculated by

**Figure 4:**
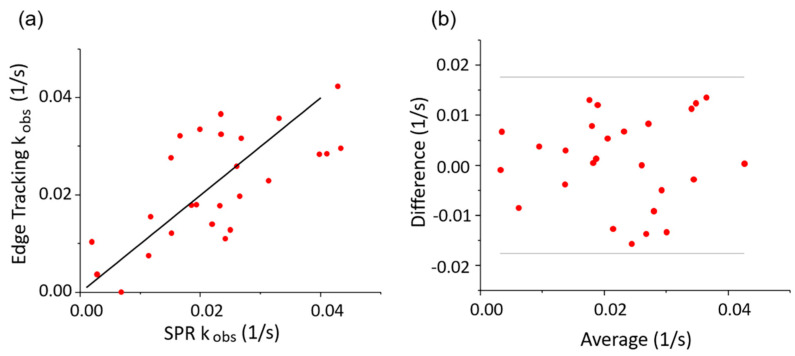
(**a**) Plot of the observed kinetics constant from edge tracking and the SPR method for simultaneously measured cells. Each dot represents a single cell that has both SPR data and edge tracking data for the same binding events. The solid black line represents the expected one-to-one ratio. (**b**) The Bland–Altman plot shows the discrepancies between SPR and the edge tracking algorithm in more detail. The y-axis represents the *k*_obs_ difference between the two methods (edge tracking *k*_obs_ − SPR *k*_obs_), and the x-axis represents the average *k*_obs_ of the two methods ((edge tracking *k*_obs_ + SPR *k*_obs_)/2). The standard deviation of the difference between the two methods is 0.009 s^−1^. The gray lines mark plus and minus at two times the standard deviation, ± 0.018 s^−1^.

*k*_obs_ = *C*_L_ × *k*_a_ + *k*_d_, (1)

where *C*_L_ is the ligand (WGA) concentration, *k*_a_ is the association rate constant, and *k*_d_ is the dissociation rate constant. Figure 4 shows the correlation of *k*_obs_ obtained from edge tracking and SPR imaging. Figure 4a shows a linear regression with a slope of 0.97 when the interception is set to be zero, which is very close to the ideal of 1, regardless of the variation of individual data points that are caused by cell-to-cell variation. This means that the binding kinetics from edge tracking correlate well with those from SPR imaging and represent the true binding kinetics.

Bland–Altman plots (Figure 4b) are useful for giving greater detail of the discrepancy between the two methods. The difference does not increase with the observed rate constant, at least not within the tested range. This simplifies comparison and implies that the method is valid within the tested range. The standard deviation of the difference between the methods is 0.009 s^−1^, with all the data points being within ± 0.018 s^−1^. Therefore, cell-to-cell variation is more prominent than the difference between the two methods. If working with more cells and fitting an average kinetic constant, the discrepancy between the two methods would be secondary to the variation between cells.

The kinetics results from edge tracking correlate well with the SPR results. The error that exists is symmetrical, which gives the ideal one-to-one ratio seen in Figure 4a. The difference between the two methods is also consistent, which does not vary with the value of the kinetics constant and is smaller than cell-to-cell variation. Therefore, the integration of these two methods in a single device enables more accurate cross-validation of binding kinetics, providing more reliable results for molecular interaction detection.

## 4. Conclusions

Membrane protein binding kinetics are traditionally difficult to get, and the results are questionable, depending on the methods used. Here, we show two recently developed label-free, whole-cell-based methods, namely, SPR imaging and cell deformation tracking, which can be used together for in-situ and label-free measurements of outer membrane protein binding kinetics. Compared to SPR, cell edge deformation tracking can not only measure the whole-cell response (not limited to the bottom portion of the cell) but is also able to measure small-molecule binding kinetics [7,8]. This is important because most drugs are small molecules. Complementary to edge tracking, SPR imaging provides both binding kinetics validation and cell receptor density quantification. With some improvement in transmitted image quality and implementation in automation software, the SPRm 200 system could be used for routine quantification of binding kinetics of drug candidates to membrane protein drug targets with whole-cell assays by both edge tracking and SPR imaging, without the need to isolate and purify membrane proteins.

## Figures and Tables

**Table 1:** Measured WGA binding kinetic constants with edge tracking and SPR.

	Edge Tracking *k*_a_ (M^−^^1^s^−^^1^)	Edge Tracking *k*_d_ (s^−^^1^)	Edge Tracking *K*_D_ (M)	SPR *k*_a_ (M^−1^s^−1^)	SPR *k*_d_ (s^−1^)	SPR *K*_D_ (M)
**Cell1**	6.1 × 10^4^	4.4 × 10^−3^	7.21 × 10^−8^	5.5 × 10^4^	4.9 × 10^−3^	8.91 × 10^−8^
**Cell2**	6.4 × 10^4^	4.6 × 10^−3^	7.19 × 10^−8^	5.9 × 10^4^	4.8 × 10^−3^	8.14 × 10^−8^
**Cell3**	5.5 × 10^4^	3.5 × 10^−3^	6.36 × 10^−8^	4.8 × 10^4^	4.5 × 10^−3^	9.37 × 10^−8^
**Cell4**	5.3 × 10^4^	5.5 × 10^−3^	10.4 × 10^−8^	7.6 × 10^4^	5.3 × 10^−3^	6.97 × 10^−8^
**Cell5**	6.1 × 10^4^	6.5 × 10^−3^	10.7 × 10^−8^	7.7 × 10^4^	5.8 × 10^−3^	7.53 × 10^−8^
**Cell6**	6.4 × 10^4^	2.6 × 10^−3^	4.06 × 10^−8^	5.3 × 10^4^	4.1 × 10^−3^	7.74 × 10^−8^
**Cell7**	5.4 × 10^4^	4.3 × 10^−3^	7.96 × 10^−8^	6.8 × 10^4^	4.9 × 10^−3^	7.21 × 10^−8^
**Mean**	(5.9 ± 0.47) × 10^4^	(4.5 ± 1.27) × 10^−3^	(7.69 ± 2.29) × 10^−8^	(6.2 ± 1.15) × 10^4^	(4.9 ± 0.54) × 10^−3^	(7.98 ± 0.89) × 10^−8^

Note: *k*_a_ is association rate constant, *k*_d_ is dissociation rate constant, and *K*_D_ is dissociation constant.
